# Recent Developments in Enzymatic Antioxidant Defence Mechanism in Plants with Special Reference to Abiotic Stress

**DOI:** 10.3390/biology10040267

**Published:** 2021-03-26

**Authors:** Vishnu D. Rajput, Harish, Rupesh Kumar Singh, Krishan K. Verma, Lav Sharma, Francisco Roberto Quiroz-Figueroa, Mukesh Meena, Vinod Singh Gour, Tatiana Minkina, Svetlana Sushkova, Saglara Mandzhieva

**Affiliations:** 1 Academy of Biology and Biotechnology, Southern Federal University, 344090 Rostov-on-Don, Russia; tminkina@mail.ru (T.M.); terra_rossa@mail.ru (S.S.); msaglara@mail.ru (S.M.); 2 Department of Botany, Mohan Lal Sukhadia University, Udaipur, Rajasthan 313001, India; mukeshmeenabhu@gmail.com; 3 Centro de Química de Vila Real, Universidade de Trás-os-Montes e Alto Douro, Quinta de Prados, 5000-801 Vila Real, Portugal; 4 Key Laboratory of Sugarcane Biotechnology and Genetic Improvement (Guangxi), Ministry of Agriculture and Rural Affairs/Guangxi Key Laboratory of Sugarcane Genetic Improvement/Sugarcane Research Institute, Guangxi Academy of Agricultural Sciences, Nanning 530007, China; drvermakishan@gmail.com; 5 Centre for the Research and Technology of Agro-Environment and Biological Sciences, Universidade de Trás-os-Montes e Alto Douro, Quinta de Prados, 5000-801 Vila Real, Portugal; lavhere@gmail.com; 6 Laboratorio de Fitomejoramiento Molecular, Centro Interdisciplinario de Investigación para el Desarrollo Integral Regional Unidad Sinaloa (CIIDIR-IPN Unidad Sinaloa), Instituto Politécnico Nacional, Blvd. Juan de Dios Bátiz Paredes no. 250, Col. San Joachín, C.P., 81101 Guasave, Mexico; labfitomol@hotmail.com; 7 Amity Institute of Biotechnology, Amity University Rajasthan, NH 11C, Kant Kalwar, Jaipur 303002, India; vinodsingh2010@gmail.com

**Keywords:** antioxidant enzymes, reaction mechanism, stressors, reactive oxygen species, secondary metabolites

## Abstract

**Simple Summary:**

Higher plants face a variety of stress conditions. There are a number of different antioxidant enzymes that help plants to cope with these stresses. During stresses, SOD catalyses the removal of •O_2_^−^ by dismutating it into O_2_ and H_2_O_2_. The CAT converts the H_2_O_2_ into water and O_2_. POX works in the extracellular space for scavenging H_2_O_2_. Plant GPX catalyses the reduction of H_2_O_2_ and HO_2_ to water and lipid alcohols, respectively. GR catalyses the reduction of oxidised glutathione (GSSG; dimeric) to reduced glutathione (GSH; monomeric). APX utilises ascorbate as a specific electron donor to scavenge H_2_O_2_ to water.

**Abstract:**

The stationary life of plants has led to the evolution of a complex gridded antioxidant defence system constituting numerous enzymatic components, playing a crucial role in overcoming various stress conditions. Mainly, these plant enzymes are superoxide dismutase (SOD), catalase (CAT), peroxidase (POX), glutathione peroxidase (GPX), glutathione reductase (GR), glutathione S-transferases (GST), ascorbate peroxidase (APX), monodehydroascorbate reductase (MDHAR), and dehydroascorbate reductase (DHAR), which work as part of the antioxidant defence system. These enzymes together form a complex set of mechanisms to minimise, buffer, and scavenge the reactive oxygen species (ROS) efficiently. The present review is aimed at articulating the current understanding of each of these enzymatic components, with special attention on the role of each enzyme in response to the various environmental, especially abiotic stresses, their molecular characterisation, and reaction mechanisms. The role of the enzymatic defence system for plant health and development, their significance, and cross-talk mechanisms are discussed in detail. Additionally, the application of antioxidant enzymes in developing stress-tolerant transgenic plants are also discussed.

## 1. Introduction

Plants are immobile; they cannot escape from biotic, (i.e., pathogens, parasites, grazing) and abiotic (such as drought, flooding, salinity, low-high temperatures, ultraviolet radiation, nutrient deficiency, heavy metal (HM) toxicity) stresses. Plant growth, development and productivity are influenced by a variety of environmental stresses. These stresses often perturb the homeostasis and ion distribution in plant cells and induces osmotic stress, leading to an enhancement in the accumulation of reactive oxygen species (ROS) [1]. The production and accumulation of ROS in the plants result in severe destruction of cell organelles and functions cause membrane peroxidation, leading to damage in the cell membrane, degradation of biological macromolecules and ultimately cell death. The ability of plants to scavenge the toxic effects of ROS seems to be the most important determinant for their tolerance to different stresses. Antioxidants are the first line of defence against the damages caused by free radicals and are critical for the optimum health of plant cells [2,3,4,5]. Plant antioxidants play a significant role in assisting plant development through a wide variety of mechanisms and functions. 

There are several antioxidant enzymes associated with ROS scavenging in plants, and the synthesis of these enzymes is known to be enhanced during the exposure to oxidative stresses [6]. The ROS comprise free radicals, such as superoxide radicals (•O_2_^−^), hydroxyl radicals (•OH), perhydroxyl radicals (HO_2_^−^) and alkoxy radicals, and non-radical forms, i.e., hydrogen peroxide (H_2_O_2_) and singlet oxygen (^1^O_2_), present in the intra- and extra-cellular locations of the plant. Superoxide radicals (•O_2_^−^) can be generated by a single electron transfer (e^−^) to dioxygen (O_2_). 

Chloroplasts and mitochondria are the two main sites for the generation of ROS. The photosynthetic electron transport system (ETS) is one of the important sites for the generation of ROS, and this site has the potential to generate singlet oxygen ^1^O and superoxide (•O_2_^−^). Plant mitochondria differ from animal as it possesses O_2_ and carbohydrate-rich environment [7], and also being associated with photorespiration. The mitochondrial ETC (mtETC) is also a source of generation of ROS as it houses sufficiently energised electrons to reduce the O_2_. The major parts of the mtETC responsible for producing ROS are Complex I and Complex III [8]. Other sources of ROS production in the mitochondria are from the different enzymes present in the matrix. There are other sites as well for the generation of ROS, such as the endoplasmic reticulum, cell membrane, cell wall and apoplast. 

Evolution has equipped plants with a wide range of defence measures, which include various enzymatic strategies to scavenge free ROS in plant cells [9,10]. The tolerance mechanisms in stressed plant include a number of physio-biochemical strategies, which includes many enzymatic components, such as superoxide dismutase (SOD), catalase (CAT), peroxidases (POX), glutathione peroxidase (GPX), glutathione reductase (GR), glutathione S-transferases (GST), ascorbate peroxidase (APX), monodehydroascorbate reductase (MDHAR) and dehydroascorbate reductase (DHAR), and non-enzymatic components, such as ascorbic acid (AA), glutathione (GSH), phenolic compounds, alkaloids, flavonoids, carotenoids, free amino acids and α-tocopherols [11,12,13]. However, in the present review, we have exclusively focussed on the role and mechanisms of enzymatic components in the plant to scavenge the ROS and to cope with the stress conditions. These enzymes are selected on the basis of majority of the research reports available and with their proven utility in transgenic plants to cope with the stress conditions (Table 1). During stresses, SOD catalyses the removal of •O_2_^−^ by dismutating it into O_2_ and H_2_O_2_, CAT converts the H_2_O_2_ into water and molecular oxygen (O_2_) and POX works in the extra-cellular space for scavenging H_2_O_2_. Plant GPX catalyses the reduction of H_2_O_2_ and HO_2_ to water and lipid alcohols, respectively, using thioredoxin as an electron donor. Glutathione reductase catalyses the reduction of oxidised glutathione (GSSG; dimeric) to reduced glutathione (GSH; monomeric) and APX utilises ascorbate as specific electron donor to scavenge H_2_O_2_ to water.

These enzymes not only protect various components of the cells from damages, but also play an important role in plant growth and development by modulating cellular–sub-cellular processes such as mitosis [14], cell elongation [15], senescence [16] and cell death [17], and are also involved in a wide range of processes, such as cell differentiation [18], cell growth/division [19], regulation of senescence and sulphate transport [20,21], detoxification of xenobiotics [22], conjugation of metabolites [23], regulation of enzymatic activities [24], synthesis of proteins and nucleotides [25,26], phytochelatins [27] and expression of stress responsive genes [28]. The antioxidant defence system protects the unsaturated membrane lipids, nucleic acids, enzymes and other cellular structures from the negative impacts of free radicals [29]. Therefore, the antioxidant defence system of plants has been attracting considerable interest of the scientific community [29,30].

The aim of this review is to critically analyse and to comprehend the state-of-art knowledge about the reaction mechanism of the different enzymatic antioxidants defence systems to cope with the various stresses faced by the plants. The role and mechanism of SOD, CAT, POX, GPX, GR, GST, APX, MDHAR and DHAR enzymes and their recent molecular understanding are discussed. Furthermore, the cross-talk between different enzymatic components are highlighted to decisively understand the overall physiological state of the plants during encountered stress. Lastly, the utility of these enzymes in developing stress-tolerant transgenic plants are also discussed.

## 2. Enzymatic Antioxidant Defence Systems in Plants

**Figure 1:**
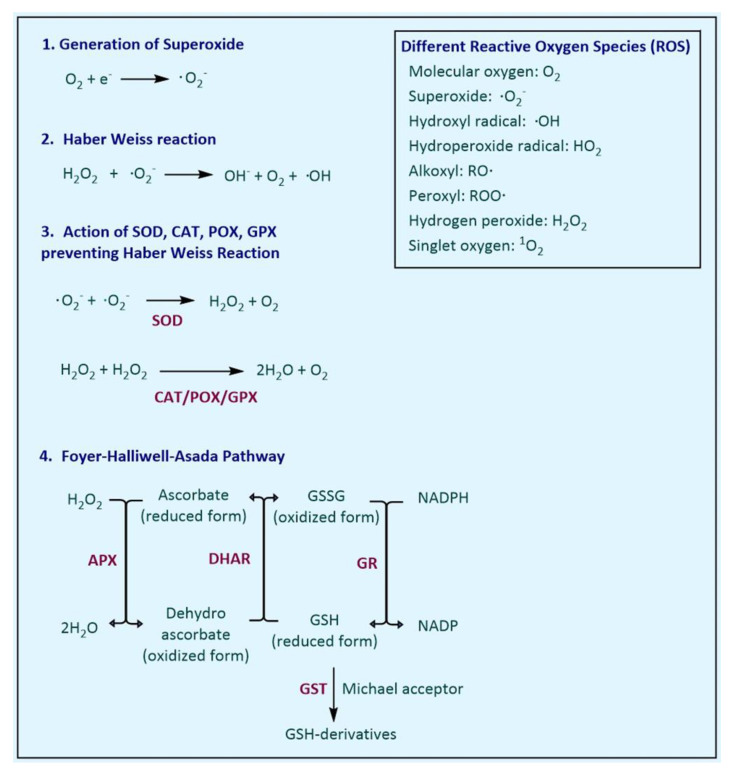
Basic reactions catalysed by the antioxidant enzyme system.

The antioxidant defence system in the plant comprises several different enzymes. They are mainly involved in either preventing the Haber-Weiss reaction (Figure 1) or the Foyer–Halliwell–Asada pathway, which reduces the H_2_O_2_ and utilises the reducing potential of NADPH. In the following section, the reaction mechanism of SOD, CAT, POX, GPX, GR, GST, APX, MDHAR and DHAR enzymes, along with their molecular characteristics, are discussed in detail.

### 2.1. Superoxide Dismutase

Superoxide dismutase (SOD; EC 1.15.1.1) catalyses the dismutation of superoxide anion (•O_2_^−^) to form H_2_O_2_ and O_2_ (Figure 1). Dismutation reactions are the reactions in which both oxidation and reduction reactions take place in the same reactant (•O_2_^−^, in this case) in a biological system, ultimately yielding two compounds: one of a higher oxidation state (such as O_2_) and another of a lower oxidation state (H_2_O_2_, as in this case). This enzyme is considered as one of the major enzymatic systems to scavenge stress-generated free radicals •O_2_^−^ in the plants [31]. Other enzymes, such as CAT and POX, work in close synchrony with SOD to prevent formation of more harmful ROS by both •O_2_^−^ and H_2_O_2_ through a Haber-Weiss reaction (Figure 1). 

Depending upon the metallic co-factors (such as Cu, Zn, Mn, Fe and Ni) that are associated with SOD, it has different isoforms, such as Cu/Zn-SOD, Mn-SOD and Fe-SOD [32]. Ni-SOD is reported in bacteria and cyanobacteria; however, it has not yet been reported in higher plants [12]. The gene sequence of Ni-SOD enzymes is also different from other SODs [33]. The Cu/Zn-SOD isoform present in cytoplasm, peroxisomes, chloroplast and at extracellular location (apoplast) and Fe-SOD in the chloroplast of the plants, whereas Mn-SOD is found in the matrix of the mitochondria and in peroxisomes [34].

Superoxide dismutase is a large protein consisting of two main domains. The secondary structures that are present include α-helices and β-sheets [35]. It is reported that Cu/Zn-SOD is dominated by β-strands, while Mn- and Fe-SOD are dominated by α-helices followed by β-strands [36]. Sequence alignment and structural and evolutionary study revealed homology between Mn- and Fe-SODs, whereas Cu/Zn-SOD was observed to be distinctly related to either of Mn- or Fe-SOD [36]. There are two pathways for final protein formation of Cu/Zn-SOD. The Cu-Chaperone for SOD (CCS)-dependent pathway requires Cu-chaperone to covalently attach to the Cu ion, thereby activating the SOD, while another pathway is CCS-independent [37].

The Cu/Zn-SOD in its native form is a homo-dimer (cytosolic) and homo-tetramer (chloroplast and apoplast); similarly, Mn-SOD can also exist as a homo-dimer or homo-tetramer in peroxisome and mitochondria [38,39]. The side chain of aspartate and histidine, present in the two-domain, forms the metal binding site, while in chloroplastic Fe-SOD, three histidine and one aspartic acid donor groups serve as a tetradentate ligand and stabilises the bound Fe to the active site of the enzyme [40].

The role of these co-factors is to stabilise the transitional bond formation during metabolising intermediates. Copper, Zn, Mn and Fe all have +2 valency due to the corresponding stable electronic configuration (ions formed by losing two electrons). Superoxide (•O_2_^−^) has one extra electron that can be transferred to these co-factors during the formation of the reaction intermediate. Ultimately, these excess electrons are combined with H to yield H_2_O_2_ as the end product and eventually releasing O_2_. The affinity of bound co-factors for single charged anions, such as F^−^, CN^−^ and N_3_^−^, is the reason why these SOD enzymes are competitively inhibited by anions. In Cu/Zn-SOD, Zn has role of giving structural stability to the enzyme per se rather than contributing any functional attribute to the catalytic activity [41].

Recently, genome-wide association studies (GWAS) have attracted the attention of scientist to understand the variation in traits with respect to single nucleotide polymorphisms (SNPs). Employing the GWAS approach, it was observed that *Oryza sativa* have eight SOD coding genes, while nine SOD coding genes were observed in *Arabidopsis thaliana* genome [42,43]. Furthermore, it was revealed that distribution of various isoforms of SODs are on five different chromosomes in *O. sativa* and on all five chromosomes in *A. thaliana*. Likewise, previous studies reported 18 loci in the *Gossypium* spp. genome [44,45]. Recently, a total of 10 SOD genes were reported in *Camellia sinensis* genome, including seven for Cu/Zn-SODs, two for Fe-SODs and one Mn-SOD [46]. Similarly, 13 SOD genes were identified in the *Vitis vinifera* genome, and expression studies revealed that few of these genes are involved in *V. vinifera* berry ripening [47,48]. Additionally, 11 SOD genes were identified using GWAS approach in *Pyrus bretschneideri*, while two genes, *PbrCDS5* and *PbrFSD1*, were picked out as the candidates postulated in postharvest ripening of *P. bretschneideri* [49]. 

Few studies have reported regulatory aspect of SOD genes in some plants. One transcription factor (CsPIF8), known to interact with phytochrome genes, has been found to upregulate the SOD expression in *Citrus*, thereby contributing to cold tolerance. CsPIF8 is reported to bound directly to the E-box (CANNTG) of the SOD promoter [50]. Recently, the role of microRNA in gene regulation has emerged. In this line, it was observed that one microRNA, namely, csn-miR398a-3p-1, directly cleaves Cu/Zn-SOD mRNA in the *C. sinensis*, thereby negatively regulating the gene expression of Cu/Zn-SOD [46]. Moreover, it was also observed that microRNA negatively regulates the antioxidant enzymes in the *Zanthoxylum bungeanum* [51].

Enhanced SOD activity in response to the water deficiency was detected in various *Phaseolus vulgaris* cultivars [52] and *O. sativa* [53,54]. The SOD activity was found to be higher in *Trifolium repens* leaves during limited water irrigation [55], and saline stress in *Cicer arietinum* [56] and *Solanum lycopersicum* [57]. Activity of all three isoforms of SOD have been found to increase the tolerance response to saline condition of *C. arietinum* [58]. Transgenic *A. thaliana* over-expressing Mn-SOD significantly increased salinity tolerance [59]. In the field condition, supplemental ultraviolet-B enhanced SOD activity in *T. aestivum* and *Munga radiata*, and caused various responses among *Glycine max* cultivars [60]. 

### 2.2. Catalase

Catalase (CAT; EC 1.11.1.6) does not require any reductant for its catalytic activity, as in this two-step reaction, H_2_O_2_ first oxidises the Fe present in the CAT, making an intermediate iron peroxide known as compound I. The enzyme can remain in this resting-state if the concentration of H_2_O_2_ is low. However, at higher H_2_O_2_ concentration, the second molecule of H_2_O_2_ serves as a reductant for this intermediate compound I, thereby regenerating enzyme and releasing water and oxygen in the second step. Moreover, since the formation of compound I changes the absorption spectra of the enzyme, it can be monitored in vivo as well as in vitro [61].

Step 1: CAT-Fe-OH + H_2_O_2_ → CAT-Fe-OOH + H_2_O

(Iron peroxide; compound I)

Step 2: CAT-Fe-OOH + H_2_O_2_ → CAT-Fe-OH + H_2_O + O_2_

Hydrogen peroxide is water soluble and has a long half-life in comparison to the other ROS. The sources of H_2_O_2_ in the plant cell are the chloroplast, mitochondria, peroxisome and apoplastic regions. In all of these compartments, H_2_O_2_ is synthesised after dismutation of •O_2_^−^ by SOD. Furthermore, H_2_O_2_ is generated by glycolate oxidase in peroxisome [62]. Although at lower concentrations, H_2_O_2_ serves as a secondary messenger in plant development, stress sensing [63,64], fruit ripening and postharvest events [65], a slight increase in its concentration can be deleterious due to its involvement in the Haber-Weiss reaction. Therefore, antioxidant enzyme achieves the delicate balance of maintaining low micro-level concentrations of H_2_O_2_. CAT and APX help the plants to cope with H_2_O_2_-induced cellular damage. Catalase has a high K_m_ value for H_2_O_2_ in comparison to APX. Therefore, CAT is more active at a high concentration of H_2_O_2._ As APX is present at multiple sub-cellular locations, it fine tunes the scavenging activity [65]. Due to the presence of Fe in CAT, it can be inhibited by cyanide, azide and hydroxylamine. Inhibition by aminotriazole and mercaptoethanol shows the participation of a thiol group in the catalytic site of the enzyme [66].

Catalase is a tetrameric biomolecule in its native form, consisting of four subunits ranging between 54 to 59 kDa, together making a molecular weight of about 240 kDa. However, CAT of 320 kDa (dimer of 160 kDa subunits) is also reported in *Mesembryanthemun crystallinum* [67]. Likewise, a homodimer of 125–135 kDa, consisting of a 55-kDa monomer unit, has been reported in *Capsicum annuum* [68]. 

Multiple forms of CAT enzyme have been reported, and they are expressed at different developmental phases in different tissues of plants [66]. Phylogenetic and synteny analyses revealed that the duplication of primordial gene, polyploidy events and differential loss of introns contributed to the increase in CAT gene numbers to three in monocots. Three different classes have been assigned, i.e., Class I, II and III, based on the *Nicotiana* *tabacum* genes. Class I CATs have a role in photosynthetic tissues and are involved in scavenging of H_2_O_2_ generated during photorespiration; Class II is prominent in vascular tissues and lignification in response to ABA and during senescence, while Class III is expressed in seeds and reproductive tissues and activity is high during catabolism of fatty acids during glyoxylate cycle in glyoxysomes. Three isoforms have been identified in *Zea mays*, namely, CAT1, CAT2 and CAT3. These forms are identical in their coding region and differ only in variable intron segments. Furthermore, the promoter sequence is also different in these different isoforms [32]. Similarly, *Arabidopsis* genome also has three CAT genes. Recently, seven CAT genes were identified in *Gossypium* using GWAS [69]. The CAT gene numbers varies in different plants, e.g., three in *O. sativa*, *C. pepo* and *Cucumis sativus*, two in *Hordeum* spp. and one in *Ipomoea batatas*, *Ricinus communis* and *S. lycopersicum* [69].

It has also been suggested that the CAT gene family is regulated at multiple levels, e.g., at transcription level by transcription factors (TFs) and at post-transcriptional level by alternative splicing and mRNA sponging [69]. In one study, TaMIR1119 (an miRNA family member of *T. aestivum*) overexpressing lines of *N. tabacum* were developed, showing increased activity of SOD, CAT and POD subjected to limited water supply [70]. The CAT activity was found higher in sensitive cultivars of *T. aestivum* under water stress [71], and *C. arietinum* also exhibited upregulated CAT activity in leaves and roots during salinity stress [56,58]. The *O. sativa* mutant HTT-121 is identified as heat-tolerant and exhibited increased CAT activity, in comparison to the heat-sensitive mutant [72]. Interestingly, a recent report suggested decreased CAT activity due to photoperiod stress in *A. thaliana* [73]. Moreover, melatonin (N-acetyl-5-methoxytryptamine) was found to boost CAT activity in root under drought in two contrasting genotypes of *Brassica napus*, Qinyou8 (drought-sensitive) and Q2 (drought-tolerant), indicating regulation of CAT by melatonin [74]. These findings suggested many unexplored aspects of CAT enzyme regulation, and hence, this needs thorough investigation.

### 2.3. Peroxidase

Plant peroxidases (POX; EC 1.11.1.7) are the only Class III peroxidase enzymes that work in the extracellular space for scavenging H_2_O_2_. Other classes are Class I, which includes cytochrome c POX, CAT-POX and APX, and Class II, which includes Mn-dependent POX, and ligninase [75]. These three classes are established only for the plant POX family. Peroxidase are glycoproteins that are synthesised via the endoplasmic reticulum and Golgi apparatus route, leading to their secretion to either the extracellular space or to the vacuoles. In native form, POX functions as a single polypeptide in the majority of plants, with 300–350 amino acid residues having 33–55 kDa MW. However, exceptions have also been reported, such as homo-tetramers in *Cocos nucifera* [76], homodimers in *B. oleraceae* [77] and heterotrimers in *Leucaena leucocephala* [78]. The POX characterised from *Fagopyrum esculentum* revealed two isozymes, i.e., POX I and POX II, with 46.1 and 58.1 kDa MW, respectively, and optimum activity temperature of 30 °C (POX I) and 10 °C (POX II), respectively [79]. Guaiacol peroxidase (GOPX) is a POX enzyme, which utilises guaiacol (o-methoxyphenol) as a common reducing substrate for its H_2_O_2_-dependent oxidation [80]. 

**Figure 2:**
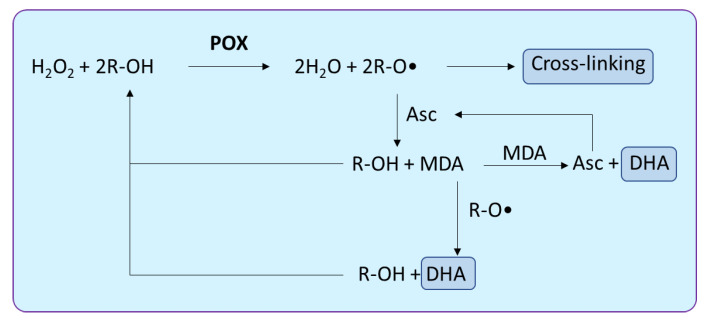
Reaction catalysed by peroxidase (POX) and cascading events; R = any phenolic compound, Asc = Ascorbate, MDA = Mono-dehydro-ascorbyl radical, DHA = de-hydro-ascorbate. When compared with the antioxidant activity of different enzymes under HM treatment, it was found that SOD and CAT activity were inhibited at high dose treatment of arsenite (AsIII) and arsenate (AsV) in *Lemna valdiviana*, while POX activity remained unchanged under increasing concentration of these HM treatments, which indicates that some of the antioxidant enzymes remain functional [84]. The importance of having different enzymes, employed by the plant to cope with the stress conditions, is highlighted in that study. Furthermore, stress memory in plants plays an important role. To understand the correlation between the stress memory and activity of POX (among other antioxidant enzymes), *Alopecurus pratensis* was given intermittent drought treatment, and it was found that drought memory is linked to higher levels of antioxidative enzymes, including POX, SOD and CAT [85]. Recently, a paper posted on a pre-print server (not peer reviewed) revealed 47 POX genes in the grapevine genome, classified into seven subgroups based on their phylogenetic analysis and GWAS approach [86]. After studying horseradish POX under in vitro conditions, three different reaction mechanisms catalysed by POX were proposed by Jovanović et al. [87], i.e., Peroxidatic, oxidative and hydroxylic cycles, which are described in Figure 3.

In terms of antioxidant mechanism, ascorbate-glutathione are the leading molecules that maintain the redox homeostasis via the Foyer-Halliwell-Asada Pathway, while flavonoids, phenolic compounds and POX serve as the second line of defence system that helps plants to cope with excess H_2_O_2_ [81,82]. However, POX is involved in a diverse role in plant growth and development. Apart from its role in the catabolism of H_2_O_2_ and redox homeostasis, this family of enzymes is involved in cell wall cross-linking, cell wall loosening, lignification, suberisation and auxin catabolism. The POX scavenges the H_2_O_2_ by catalysing the oxidation of phenolic substrates using H_2_O_2_ as an electron acceptor. Subsequent reactions led to the generation of MDA (mono-dehydro-ascorbyl radical), ascorbate and DHA (de-hydro-ascorbate) and cross-linking product of phenolic compounds, such as lignin or suberin (Figure 2). Such types of enzymatic cross-linking reactions are an important way to study the protein–protein interaction, and in a recently conducted study, POX from *Z. mays* and *P. vulgaris* roots were isolated and found functional for cross-linking of globular protein patatin from *Solanum tuberosum*. Phenolic compounds were found helpful in the cross-linking reaction [83]. This study provides insights into the utility of POX in understanding the biophysical structure of the protein of interest. 

### 2.4. Glutathione Peroxidase

**Figure 4:**
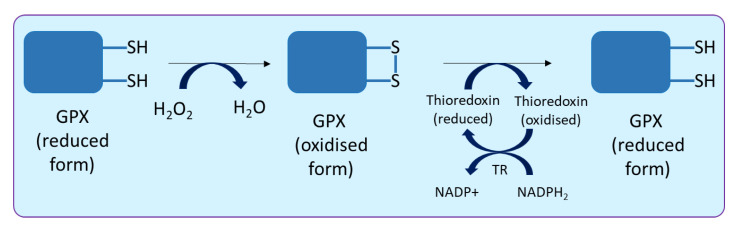
Reaction mechanism of glutathione peroxidase (GPX).

Glutathione (GSH) is derived from three amino acids, i.e., glutamate, cysteine and glycine. During its synthesis, firstly, a bond is made between glutamate and cysteine, known as gamma peptide linkage, followed by the addition of glycine to the C-terminus of glutamyl-cysteine, ultimately forming tripeptide glutathione (γ-glutamyl-cysteinyl-glycine). Glutathione exists in two different states, i.e., reduced (GSH) and oxidised (GSSG). Glutathione peroxidase (GPX; EC 1.11.1.9) catalyses the reduction of H_2_O_2_ and HO_2_ to water and lipid alcohols, respectively. In plants, this enzyme is a thiol-based (an organic compound containing the -SH group) enzyme and uses thioredoxin as an electron donor to palliate damaging impact of H_2_O_2_ (Figure 4).

The reaction occurs in three steps, (a) the oxidation of cysteine residue to sulfenic, (b) the formation of disulphide bond with the second cysteine present in the enzyme and (c) the reduction of disulphide bond using thioredoxin. Thioredoxins are a small redox protein (12 kDa), present in all organisms, and they facilitate reduction of GPX by cysteine thiol-disulphide exchange. Oxidised thioredoxin is regenerated after a reduction by thioredoxin reductase (TR) using NADPH_2_. There are two main differences between plant and animal GPX; first, Plant GPX contains cysteine in their active site, whereas, in the majority of the metazoans, seleno-cysteine is present in the active site [88]; second, for the regeneration of oxidised GPX, thioredoxin is used in the case of plants, while, in animals, regeneration occurs via GSH. Noticeably, Se containing GPX has also been demonstrated in *Aloe vera*. In this plant (*A. vera*), GPX is reported to consist of four subunits of 16 kDa each, with one selenium atom per subunit, as found with most GPX from animal sources [89]. In addition, glutathione transferase enzymes having GPX activity have also been reported [90]. Different forms of GPX (different enzymes having GPX activity) are now designated as GPX-like (GPXL), to avoid confusion in the nomenclature [91]. The activity of GPX has been implied as a biomarker to manifest intracellular oxidative stress [92]. An antioxidant enzyme inhibitor isoproturon was studied to understand the magnitude of inhibition against GSH-associated enzymes in *T. aestivum*, and the highest level of inhibition was observed in the GPX activity, followed by γ-glutamyl-cysteine synthetase (γ-GCS), glutathione synthetase, glutathione-S-transferase and glutathione reductase activities [93]. 

The higher GPX activity was reported during various abiotic and biotic stresses [11,94,95,96,97,98]. Roychoudhury et al. [99] noted that antioxidative enzymes activities such as GPX and APX are enhanced in salt-sensitive and -tolerant varieties of *O. sativa* under Cd stress; however, the activity was excessively increased in salinity tolerant cultivars. The GPX gene from a medicinal plant *Rhodiola crenulate* is transferred to *Salvia miltiorrhiza* under a strong constitutively expressed promoter CaMV 35S. Transgenic plants were found to have increased tolerance to the drought and oxidative stress [100]. The participation of GPX in response to the biotic stresses are supported by the observation that activity of two GPXs were increased under the compatible interaction of *Plasmopara halstedii* and *Helianthus annuus,* whereas they were decreased under the incompatible interaction with an a virulent strain [101]. Similar findings have been observed during the rice-blast pathogen interaction [102]. During the hypersensitive response, the burst in ROS is one of the important factors impeding growth of the biotrophic pathogens [103], whereas it was shown to facilitate the infection of necrotrophic pathogens [104]. Wounding and pathogenic infections were shown elsewhere to be associated with ROS and ROS recycling enzymes [105,106]. It was revealed that the ROS, especially H_2_O_2_, accumulate following the internode rubbing, and are considered as the key signalling factors in the thigmo-morphogenetic response [107,108,109]. These observations again highlight the dual effects of ROS on the living organisms, therefore, acting both as cell-death executioners as well as in pro-survival signalling cascades [110,111].

### 2.5. Glutathione Reductase

Glutathione reductase (GR; EC 1.8.1.7) is a flavo-protein oxidoreductase NAD(P)H-dependent enzyme, and an important component of ascorbate-glutathione pathway. The GR catalyses the reduction of oxidised glutathione (GSSG; dimeric) to reduced glutathione (GSH; monomeric). It has been characterised in a number of plants, and reported that it occurs in its native form as homodimers (with 100 to 150 kDa MW), containing one flavin adenine dinucleotide (FAD) per monomer. Two cysteine residues are present at the active site of GR [89,112,113,114,115]. The reaction catalysed by GR befalls in a ping-pong fashion and occurs in two steps: in the first step, the FAD is reduced by NADPH. The reduced FAD transfers the reduction power to the thiol group on cysteine in the active site. In the second step, the reduction of GSSG occurs via a thiol-disulphide interchange reaction, which involves a nucleophilic attack on the disulphide bond of GSSG, reducing the dimeric GSSG to GSH [116]. Cysteine is the first amino acid produced from sulphur assimilation, and it has been reported that GR plays a major role in sulphur assimilation in the plants, by supporting the activity of adenosine 5′ phosphosulphate reductase (APR). GR recycles the GSSG back to GSH, which serves as an electron donor for the APR activity [117], creating a positive feedback loop for sulphur assimilation.

The majority of the activity (80%) of GR is reported from chloroplast in photosynthetic tissues; however, its presence has also been reported in cytosol, nucleus, peroxisomes and mitochondria [116]. In *Arabidopsis*, the GR proteins were classified into two classes due to the presence of N-termination signal sequence, GR1 and GR2. GR1 is shorter enzyme reported to be present in cytosol, nucleus and peroxisomes, while GR2 contains the transit sequence required for targeting to the chloroplast and mitochondria [118]. The crucial role of chloroplastic GR2 was investigated under attenuated expression (using RNA interference) of GR2 in transgenic *Arabidopsis*. It was revealed that GR2 activity is essential for photoprotection of photosynthetic machinery. Transgenic plants with reduced GR2 activity were found to be highly sensitive to excess light and Photosystem II activity is severely affected. The activity of GR2 is essential for the electron transfer at the acceptor side of PSII, and for the repair of photodamaged PSII, by preventing the accumulation of H_2_O_2_ under excess light [119]. It was observed that the role of chloroplastic GR2 is indispensable, while mitochondrial GR2 and cytosolic GR1 play additional roles in mitigating oxidative stress [120]. 

Genome-wide association studies indicated three loci of GR in the *O. sativa* genome and two loci in *Arabidopsis* genome [118]. It has been revealed that different plants have different number of isoform of GR, such as two isoforms reported in *N. tabacum* [121], *Vigna unguiculata* [122], *P. vulgaris* [123], *Pisum sativum* [124], *Brassica* [125] and *Arabidopsis* [119] and three isoform reported in *T. aestivum* [126], *Hordeum vulgare* [127], *Z. mays* [128] and *O. sativa* [129]. However, in one study, two isoforms of GR in *T. aestivum* were reported after cloning and characterisation of GR genes (*TaGR2-1* and *TaGR2-2*). *TaGR2-1*, with a 1490-bp open reading frame (ORF), encodes 496 amino acid residues with an estimated 53 kDa MW, whereas *TaGR2-2* encodes for a polypeptide of about 52.9 kDa MW. The expressions of *TaGR2* and enzymatic activity of GR were upregulated in nitrogen-starvation in *T. aestivum*. Multiple sequence alignment reveals *TaGR2* showing homology with cytosolic GR [130]. Furthermore, GR gene (*SpGR*) from *Stipa purpurea* was characterised with ORF of 1497 bp, encoding 498 amino acids. The transgenic overexpression of *SpGR* confers higher salt tolerance in *Arabidopsis* [49]. Recently, three GR genes were cloned and characterised from *Populus trichocarpa*. The *PtGR1.1* and *PtGR1.2* were found to be localised in the cytoplasm, while *PtGR2* was observed in the chloroplast [131]. 

### 2.6. Glutathione-S-Transferase

Glutathione S-transferases (GST; EC 2.5.1.18) or glutathione transferases are multifunctional enzymes that catalyse the nucleophilic attack of the sulphur atom of glutathione, leading to the conjugation of the tripeptide glutathione to electrophilic compounds (positively charged having vacant orbitals; therefore, an electron pair acceptor) or hydrophobic compounds to form more soluble peptide derivatives. This family of enzymes is quite versatile in nature, with numerous alternative substrates, covering wide ranges of reactions. The GSTs substrates have a common chemical feature, i.e., carbon-carbon double bonds near to an electron acceptor group, termed the ‘Michael acceptor’ [132]. It was first discovered in *Z. mays*, where these enzymes were found to be involved in the detoxification of herbicide atrazine via conjugation reaction [133]. With time, their roles as carriers for hormones, secondary metabolites and other enzymes have been discovered. Moreover, they are reported to be involved in biotic and abiotic stress tolerance, regulation of redox homeostasis, as well as in apoptosis [134].

The GST enzyme consists of two main domains, i.e., the N-terminal and the C-terminal. Alpha-helices and beta-strands are arranged in a thioredoxin-like fold in the N-terminal domain, while the C-terminal domain consist of only α-helices. The N-terminal contains a glutathione binding site and the C-terminal have hydrophobic substrate binding site. A short linker sequence of about 10 residues connects both the domains. Overall, the GSTs protein family is divided into 36 classes, which include plants, animals, fungi and bacteria; 14 classes have been recognised among eukaryotic photosynthetic organisms. Recently, 10 classes were observed in plants and these are: GSTU (Tau; τ), GSTF (Phi; φ), GSTL (Lambda; λ), GSTT (Theta; θ), GSTZ (Zeta; ζ), DHAR (de-hydro-ascorbate reductase), TCHQD (tetra-chloro-hydro-quinone dehalogenase) and EF1Bγ (elongation factor 1B, hemerythrin and Iota; ι). Out of these, Tau, Phi, Zeta, Theta and TCHQ classes contain serine residue at the active site of GST. The rest of them contain cysteine residue at the active site in the GST. 

Glutathione transferases have been identified in a number of plants. For example, *G. max*, *A. thaliana*, *O. sativa*, *H. vulgare*, *T. aestivum*, *Populus*, *V. radiata* and *Medicago* contain 101, 55, 82, 84, 52, 81, 44 and 73 GSTs, respectively. An extensive survey on the types and functions of GSTs has been reviewed recently [133,134]. Three GSTs from *P. yatungensis* and *P. euphratica* were overexpressed in *A. thaliana*, resulting in an increased salt and drought resistance in the transgenics. This study found that the overexpression of GST reduces the accumulation of H_2_O_2_ and MDA (mono-dehydro-ascorbyl) from oxidative damage and helps to maintain the GSH/GSSG ratio under salt stress [135]. 

**Figure 5:**
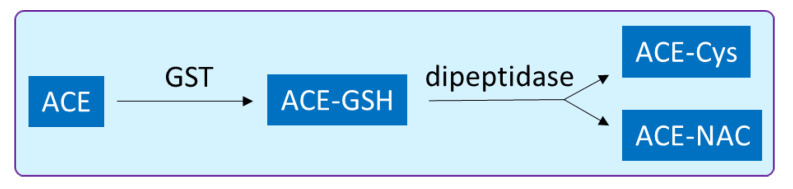
Biotransformation of acetaminophen (ACE) leading to formation of cysteine (Cys) and N-acetylcysteine (NAC) conjugates (adapted from Sun et al. [139]).

*C. sinensis* contains a number of flavonoids which are responsible for the characteristic taste of tea beverages; these compounds are stored in vacuoles. A recent study revealed that three GST genes, namely *CsGSTa*, *CsGSTb* and *CsGSTc*, are responsible for the storage of anthocyanins, flavonols and proanthocyanins in *C. sinensis* cells [136], and it also confirms the diverse function of GST enzymes in the plant cell. Heavy metal stress result in the generation of ROS in the cell, and GSTs are presumed to protect plants against HM stress through a conjugation of metal ion with glutathione. To understand how the HM interacts with GST, a molecular level interaction was studied between *Arabidopsis* GST (*AtGSTF8*) and Cd ions. An interaction study revealed the structural changes in the enzyme resulting in microenvironmental modifications around the Tyr and Trp residues. A single binding site was predicted and non-covalent interactions, such as the van der Waals forces, and the hydrogen bonds were observed to be responsible for conjugation of metal ion [137]. From the ecological perspective, the resistant population of *Polypogon fugax* weeds invading the *B. napus* fields of China were investigated, and it was found that the GST gene is responsible in conferring resistance in the *P. fugax* [138]. Furthermore, the post-conjugation biotransformation was also explored, and detoxification of acetaminophen (ACE; a pharmaceutical drug) via GST in *C. sativus* was revealed; the GSH conjugates were further converted to cysteine and N-acetylcysteine conjugates. The GST activity significantly increased after the treatment with ACE, and levels of GSH were decreased by more than 55% in the roots after 48 h (Figure 5) [139].

### 2.7. Ascorbate Peroxidase

Ascorbate peroxidase (APX; EC 1.11.1.11) is class I haem-peroxidases and is also known as ascorbate (AsA)-dependent peroxidase. This enzyme functions as a scavenger of H_2_O_2_ as well as sensors of redox alteration inside plant cells [140], and is considered a key enzyme in the Foyer-Halliwell-Asada Pathway (Figure 1). It utilises ascorbate as a specific electron donor to scavenge H_2_O_2_ to water. The APXs are unstable in the absence of ascorbate and lose the activity rapidly if the concentration of ascorbate falls below 20 μM. Similarly, Fe is also essential for the activity of the APX. APX has been reported from different sub-cellular locations in the plants, such as in the cytosol [141], mitochondria [142] and chloroplast [143], and the membrane-bound organelles, such as in peroxisome [144] and glyoxisome [145]. This enzyme has been characterised from a number of plants, e.g., *C. sinensis* (57 kDa and 34 kD; [146], *N. tabacum* (Plastid APX 34 kDa; [147], *C. pepo* (Non-plastid APX 28–31 kDa; [148], *Apium graveolens* (33.16 kDa; [136] and *Actinidia deliciosa* (18.8 kDa–110.6 kDa; [149]. Ascorbate peroxidase promoters contains ABA response element (ABRE) and heat-shock element (HSE) [150]. Its activity inhibited by cyanide and azide highlighting the role of haem in peroxidase activity. Likewise, Ellman’s reagent (5,5′-dithiobis-(2-nitrobenzoic acid) or DTNB) also inhibits the APX activity, indicating the role of thiol group in enzyme activity. Reaction catalysed by APX occurs as follows:

APX
Ascorbate + H_2_O_2_→
Monodehydroascorbate (MDHA) + 2 H_2_O


    Spontaneous oxidationMDHA→
Dehydroascorbate (DHA)

During this reaction, monodehydroascorbate (MDHA) is formed as an intermediate of the reactions, but MDHA is unstable and is spontaneously converted into dehydroascorbate (DHA). An interesting research reported a bifunctional peroxidase activity where the 4-Coumarate 3-hydroxylase enzyme, which is known for its role in the lignin biosynthesis pathway, is also found to have a cytosolic ascorbate peroxidase activity and could oxidise both ascorbate and 4-coumarate at comparable rates [151].

Recently, APX (*AgAPX1*) was characterised from *A. graveolens* and the optimum temperature for its activity was observed to be 55 °C. The expression of *AgAPX1* gene was significantly increased under drought stress. The transformation with *AgAPX1* gene conferred the drought resistance in the transgenic lines of *Arabidopsis* [43]. The APX gene from the *Dioscorea alata* cv. MH1 (Yam) is cloned and transformed in *Arabidopsis* under a strong promoter. Transgenic *Arabidopsis* was found to be more tolerant to chilling and flood stress. Furthermore, it was also revealed that low expression of APX gene in *D. alata* was responsible for the low resistance to chilling and flood stress, which can be overcome by H_2_O_2_ spraying, resulting in an increased level of APX activity [152]. Similarly, transformation of APX (*Apx1*) from *A. thaliana* to *B. juncea*, under the constitutive promoter (CaMv35S), confers salinity stress tolerance in *B. juncea* by improving the antioxidative defence mechanism [150]. Ultraviolet radiation is demonstrated to enhance the activity of APX in *A. thaliana* [153]. The APX activity is significantly correlated with lead treatment in *Eichhornia crassipes* seedlings [154]. These studies highlight the important of overexpression of *APX* gene to improve the stress tolerance traits in the transgenic lines. 

A correlation was drawn between ROS level and photosynthetic activity in *Gossypium*. RNA interference technique is used to inhibit the activity of cytosolic APX, leading to an increased ROS level in the guard cells of the stomata. Increased ROS level in guard cells inhibited stomatal opening, and thereby, stopped the influx of CO_2_ and reduced the photosynthesis in the *Gossypium*. This study indicated that the importance of a single enzyme in the antioxidant system, resulting in its impact on the overall plant growth and yield-related traits, such as the single boll weight, seed weight, seed size and lint weight of the transgenic *Gossypium* lines [47].

It is well known that melatonin synthesis helps plants to cope with stress conditions. However, a direct relationship of melatonin with an antioxidant enzyme was not revealed until recently, in a study where two enzymes of melatonin biosynthetic pathway in *Manihot esculenta* (*MeTDC2* and *MeASMT2*) were observed to interact directly with ascorbate peroxidase (*MeAPX2*). It was observed that the interaction with *MeTDC2* and *MeASMT2* significantly increased the enzymatic activity of APX, in comparison to purified APX alone. This indicates that *MeTDC2–MeAPX2* and *MeASMT2–MeAPX2* interactions activate the APX activity and increase the antioxidant capacity of the plant highlighting the merit of such interactions in influencing redox homoeostasis and stress tolerance in plants [155].

Similarly, a protein–protein interaction was also reported under nitrogen starvation conditions in *C. sinensis*. It was shown that APX (*CsAPX1*) regulates the ascorbic acid metabolism by assisting the nitrogen regulatory protein P-II (*CsGLB1*) in the plant under nitrogen starvation conditions [156]. Another study suggested that the cold stress activates the enzymes of the ascorbate-glutathione cycle under catalase deactivation in *C. sativus* leaves; however, the response time of the enzyme against various environmental stresses varies amongst different isoforms of antioxidant enzymes [157].

### 2.8. Monodehydroascorbate Reductase

Monodehydroascorbate reductase (MDHAR; EC 1.6.5.4) catalyses the reduction of MDHA to ascorbate, and therefore, plays a pivotal role in maintaining a reduced pool of ascorbate in plants. MDHAR uses NADH or NADPH for the reduction of MDHA to ascorbate. Ascorbate acts as a single electron donor, and upon reduction, it is converted into a semi-oxidised form, i.e., MDHA. Disproportionation reaction can yield ascorbate and DHA from the two molecules of MDHA; alternatively, MDHAR can reduce the single molecule of MDHA to ascorbate. Activity of MDHAR has been reported in chloroplast, mitochondria, cytosol and peroxisomes from different plants [6].

NADH or NADHP
Monodehydroascorbate (MDHA)→
Ascorbate
MDHAR


The crystal structure of the MDHAR from *O. sativa* has been investigated, and it resembles other iron-sulphur protein reductases, with a unique long loop of 63–80 residues, which forms a part of the active site. Moreover, it was found that the arginine residue at the 320 position plays an important role in the substrate binding, and the tyrosine residue at the 349 position is required for the electron transfer from NAD(P)H to the bound substrate via FAD [158]. In an interesting finding, it was reported that *Arabidopsis* 12-oxophytodienoic acid reductase isoform 3 (*OPR3*), which is involved in the synthesis of jasmonic acid (JA), has a bifunctional enzymatic activity, and *OPR3* has the ability to regenerate ascorbate from monodehydroascorbate. Characterising *OPR3* revealed NADPH-dependent monodehydroascorbate reductase activities (MDHAR), apart from α, β-ketoalkene double-bond reductase activity involved in the synthesis of JA [159].

The overexpression of the *A. thaliana* MDHAR gene (*AtMDAR1*) was found to increase the tolerance against ozone, salt and polyethylene glycol stress [6]. Recently, it was reported that thioredoxins (TRXs; y type) can influence the activity of MDHAR6 (one of the plastidial isoform of the enzyme in *Arabidopsis*), and was strongly activated by TRX y2. This highlights the involvement of TRX in the electron transport chain in chloroplast; moreover, TRX-mediated regulation may have some coordination with the synthesis of NADPH-reducing power, which is used by MDHAR and also highlights the role of major thiol-based antioxidant proteins in orchestrating the overall plant defence system [160]. The activity of MDHAR in a leaf could be an important biomarker in selecting the drought-tolerant genotype of *T. aestivum* [161]. These findings can be concluded a significant positive correlation between leaf MDHAR and grain numbers and harvest index [161]. The role of MDHAR further explored in the model algae to cope with the photooxidative stress highlighting the irradiance tolerance. Overexpression of MDHAR under a strong promoter resulted in the higher tolerance and increase in viability, whereas the downregulated MDHAR, mediated by an miRNA, reduces this tolerance [162]. Such a study in plants may provide new insights, and this would be a good topic for a future research endeavour.

### 2.9. Dehydroascorbate Reductase

Dehydroascorbate reductase (DHAR) catalyses the reduction of DHA using reduced GSH, yielding ascorbic acid and oxidised glutathione (GSSH), respectively (Figure 1). Although this reduction can occur non-enzymatically as well, the reaction rate was very slow (17 nmol min^−1^) in comparison to the reaction catalysed by DHAR (20–370 μmol min^−1^ mg^−1^) [163]. If DHA is not reduced timely, it can undergo spontaneous but irreversible hydrolysis to 2,3-diketogulonic acid [164]. Therefore, the activity of DHAR is critical in maintaining the sufficient pool of ascorbate in the plant cell. Constant recycling of these small soluble antioxidants, such as ascorbate and GSH, is essential to maintain the redox potential of the cell. Recently, few reports have revealed the crystal structure and catalytic mechanism of DHAR enzyme in different plants, such as *O. sativa* (*OsDHAR1*; [165]), *Pennisetum glaucum* (*PgDHAR1*; [166]), *A. thaliana* (*AtDHAR2*; [163]) and *P. trichocarpa* (*PtrDHAR3A*; [167]). Two to four copies of DHAR in different genome have been reported, with a few additional pseudogenes also being reported [168]. 

Reaction catalysed by the DHAR is based on a ping-pong mechanism with the following three steps: (a) GSH makes a nucleophilic attack on sulphur group of Cysteine residue present at position 20 (Cys20) of the DHAR and form a disulphide bond between GSH and –SH; (b) the GSH molecule attacks the disulphide bond and is released as GSSH, leaving the Cysteine in its reduced thioldate form; (c) DHA enters the active site of the reduced DHAR enzyme and released as ascorbate. Mutational studies have shown that Cysteine at the active site is essential for the enzymatic activity [163]. The presence of Cysteine earmark can reverse the disulphide bond formation with GSH during the reaction mechanism of the DHAR. Structural elucidation of DHAR from *O. sativa* subsp. *japonica* (OsDHAR) also revealed the location of the ascorbate-binding site overlaps with the GSH-binding site with a ping-pong kinetic mechanism for electron transfer at the Cys20 active site [165]. 

DHAR was recently characterised in *Liriodendron chinense*, a woody tree species of Magnoliaceae, with about 216 amino acid length and a cytoplasm as its subcellular location. LcDHAR overexpression in *A. thaliana* exhibited a higher concentration of ascorbate and increased salt and drought tolerance [169]. Structural comparison has revealed that DHAR is quite similar to Chloride Intracellular Channel (CLIC) protein, as well as to the Omega class of Glutathione S-transferase. In fact, CLIC has been reported to exhibit low levels of DHAR activity [163,165] and DHAR has also shown transmembrane ion conductance. However, the mechanism and significance of this is not fully understood [163].

Few studies have questioned the contribution of DHAR in maintaining ascorbate level in the plant cell [170], and it was suggested to change the name of this enzyme from dehydroascorbate reductase to glutathione dehydrogenases [168]. More recently, a novel DHAR has been characterised from *S. lycopersicum*, which is induced by Beauvericin. This novel DHAR is able to reduce DHA to ascorbate, although with a lower affinity to its substrates than the classical DHAR. In addition, it was revealed that novel DHAR is part of a larger protein complex and has a multifaceted role in the plant’s defence mechanism against stress [171]. Nonetheless, additional studies are needed to understand the role of different enzymes in the antioxidant mechanism. 

## 3. Applications of Antioxidant Enzymes in Developing Stress-Tolerant Transgenic Plants

Sustainable agriculture production is a key factor in ensuring global food security. However, there are multiple stress conditions that influences the crop growth and yield. In order to overcome these stress conditions, developing stress-tolerant plants is an important step. Understanding the role of individual gene under the influence of different stress condition can be useful in developing stress-tolerant plants. The overexpression of different genes of different antioxidant enzymes has resulted in the increase in tolerance in transgenic plants to various environmental stress conditions. Several stress-tolerant genetically engineered plants have been developed in the recent past, and the significant findings of these research reports are briefly described in Table 1. The majority of these studies focussed on abiotic stress caused due to salinity, heat, chilling, drought, flood and HM, but very few reports are available on understanding the role of these enzymes to cope with biotic stress. Furthermore, in all of these studies, the gene of the antioxidant enzyme is overexpressed under a strong promoter in transgenic lines, thereby increasing the tolerance potential of the plant to stress condition. Thus, these findings are crucial for developing stress-resistant plants, and the knowledge gained will be helpful for sustained growth and productivity of various crops in variable environmental conditions.

**Table 1:** Recent studies of transgenic overexpression of different genes encoding antioxidant enzymes in enhancing stress tolerance in transgenic plants along with significant findings.

S.No.	Transgenic Plant(s)	Gene(s)/Source	Stress Condition	Significant Finding(s)	Reference
1.	Transgenic *S. lycopersicum*	FeSOD gene from *Arabidopsis*	Salt stress	Overexpression of antioxidant enzymes significantly mitigates the harmful effects of salt stress on cytoskeleton structural organisation in roots of the transgenic line cells.	[172]
2.	Transgenic *S. tuberosum*	Cu-ZnSOD (*StSOD1* gene overexpressed under CaMV 35S promoter)	Low temperature	Activity of SOD is 1.38-fold higher compared to non-transgenic lines. Furthermore, the activity of POX and CAT were also enhanced in transgenic line, signifying the fact that increasing the activity of one antioxidant enzyme can influence the activity of other defence enzymes via cross-talk.	[173]
3.	Transgenic *Citrus sps*	CsPIF8 influencing SOD gene expression	Low temperature	Phytochrome-interacting transcription factor CsPIF8 positively regulate CsSOD expression in citrus, highlighting the cross-talk between phytochrome genes and antioxidant enzymes. In this study, it is found that CsPIF8 directly bound to the E-box (CANNTG) of CsSOD promoter and activated the promoter of CsSOD.	[50]
4.	Transgenic *Arabidopsis*	*CmSOD* gene (from winter squash; *Cucurbita moschata*) and *AtSOD* gene (from *Arabidopsis*) under a ubiquitin promoter	Low temperature	Increased resistance to chilling and less oxidative injury in transgenic lines than wild type, indicating that the overexpression of *AtSOD* and *CmSOD* led to higher SOD activity in *Arabidopsis*-enhanced chilling tolerance by eliminating •O_2_^−^. Furthermore, the activity of SOD in transgenic lines is influenced by ABA, indicating the role of plant hormone in the cross-talk with enzymes of the antioxidant defence system.	[174]
5.	Transgenic *Arabidopsis*	Cu-Zn SOD gene (*SaCu/Zn SOD*), from *Sedum alfredii*	Oxidative stress due to Cadmium	Cadmium stress induces the production of ROS, leading to oxidative stress. Cd-hyperaccumulator plant *S. alfredii* is used as a source of SOD gene, resulting in enhanced antioxidative defence capacity in transgenic *Arabidopsis* plants. The *SaCu/Zn SOD* is implicated as being responsible for conferring Cd tolerance.	[175]
6.	Transgenic tobacco	Cu/Zn-SOD gene, *SiCSD* from *Saussurea involucrata*	Drought, cold and oxidative stress	Higher activities of SODs, CAT and APX are reported in transgenic lines, and SOD is found as a positive regulator in drought and cold stress by reducing oxidant injury.	[176]
7.	Transgenic *C. grandis*	The basic helix-loop-helix (bHLH) family of transcription factors (*PtrbHLH*) from *Poncirus trifoliata*	Low temperature	Transgenic plant was found to exhibit lower electrolyte leakage and malondialdehyde content after chilling stress, lower ROS levels and elevated activity of antioxidant enzymes, including CAT, POX and SOD. Interestingly, PtrbHLH was found to bind to the promoter and activate the PtrCAT gene, thereby implicated as regulating the CAT gene activity.	[177]
8.	*Manihot esculenta*	SOD (*MeCu/ZnSOD*) and catalase (*MeCAT1*)	Biotic stress (Mite *Tetranychus cinnabarinus*)	The transgenic approach led to mite-resistant traits, as survival, reproduction and development of *T. cinnabarinus* feeding on transgenic cassava is significantly inhibited. Furthermore, the activities of SOD and CAT in transgenic cassava plants damaged by *T. cinnabarinus* significantly increased. This study highlights the role of antioxidant enzymes in developing pest resistant crops.	[178]
9.	Transgenic *Ipomoea batatas*	Peroxidase gene *swpa4* in *I. batatas*	Salt stress	Overexpressing the *swpa4* gene under CaMV 35S promoter led to 3- to 13-fold higher expression in transgenic sweet potato. Transgenic plants also showed increased tolerance to salinity conditions, with 13–26% less damage than control plants. Furthermore, photosynthetic capacity and total chlorophyll contents were less severely impacted in transgenic plants.	[179]
10.	Transgenic *Arabidopsis*	Glutathione peroxidase-like 5 gene (*AtGPXL5*) from *Arabidopsis*	Salt stress	Constitutive overexpression of *AtGPXL5* led to an increase in gene expression by 17–24 times in 6-week-old plants. It results in an increase in GSH pool and more negative redox potential than wild type and increased salt tolerance.	[91]
11.	Transgenic *Arabidopsis*	*AtGR1* encoding glutathione reductase (GR) from *Arabidopsis*	Aluminium toxicity	The overexpression of *AtGR1* led to a higher GSH pool and improved ratio of GSH/GSSG, and increased aluminium tolerance, with better root growth in comparison to the wild type under aluminium stress. Increased GSH levels were found to increase the capacity of RCS detoxification, which indicates that GR overexpression contributes to the mitigating of not only ROS, but also RCS.	[180]
12.	Transgenic *O. sativa*	*OsGSTU5* (a tau class GST in *O. sativa*)	Biotic stress	Overexpression of *OsGSTU5* provided tolerance against sheath blight disease, caused by *Rhizoctonia solani*.	[181]
13.	Transgenic *Arabidopsis*	Glutathione S-transferase from *Thermosynechococcus elongatus BP-1* (*TeGST*)	Thiocyanate (SCN^−^) stress	Overexpression of *TeGST* in transgenic plant increased the tolerance to thiocyanate (SCN-) up to 5 mmol L^−1^. This approach was found to be potentially effective to enhance the phytoremediation of environmental thiocyanates.	[182]
14.	Transgenic *Arabidopsis*	Ascorbate peroxidase (*AgAPX1*) from *Apium graveolens*	Drought tolerance	Overexpression of the *AgAPX1* gene enhanced ascorbate content, antioxidant capacity and drought resistance. Furthermore, increased antioxidant capacity does not affect the growth parameters of the plant much, as a comparatively smaller decrease in the net photosynthetic rate is observed, and a high survival rate of transgenic *Arabidopsis* lines after drought is reported.	[43]
15.	Transgenic *Arabidopsis*	Ascorbate peroxidase gene (*DaAPX*) from *Dioscorea alata*	Flood/Chilling stress	This study reports the effect of different types of stress on the expression of *DaAPX*. Yam variety Minghuai 1 (MH1), when exposed to a flood situation, showed an increase in the expression of *DaAPX*; however, chilling stress did not influence the expression profile of *DaAPX*, thereby making this variety sensitive to chilling stress. However, overexpression of *DaAPX* in *Arabidopsis* led to increased tolerance towards several abiotic stress, including flooding and chilling.	[152]
16.	Transgenic *Brassica juncea*	Ascorbate peroxidase gene (*Apx1*) from *Arabidopsis*	Salt stress	Overexpression of cytosolic *AtApx1* gene increased salinity stress tolerance in *B. juncea*. APX, along with higher activity of other enzymes such as GPX, CAT and POX, maintains the ROS homeostasis and provides tolerance to the cell, greater proline accumulation, increased chlorophyll stability index and lower chlorophyll a/b ratio.	[150]
17.	Transgenic *Nicotiana tabacum*	Monodehydroascorbate reductase from *S. lycopersicum* (*SlMDHAR*)	Salt stress	Overexpression of *SlMDHAR* in transgenic tobacco is found to increase salt stress tolerance and NO accumulation and the S-nitrosyalted *SlMDHAR* levels were found to be higher in transgenic tobacco. Results suggested that *SlMDHAR* confers salt stress tolerance by probably involving the S-nitrosylation (post-translational modification of cysteine thiol by nitric oxide group) of MDHAR.	[183]
18.	Transgenic *Arabidopsis*	Monodehydroascorbate reductase (*BvM14*-MDHAR) from *B. vulgaris*	Salt stress	The MDHAR gene is constitutively expressed in *Arabidopsis*, resulting in an enhanced salt stress tolerance phenotype, with higher AsA/DHA levels than wild-type. In addition, the overexpression seedlings showed higher activities of MDHAR and DHAR and decreased cell membrane damage.	[184]
19.	Transgenic *Arabidopsis*	DHAR (*AcDHAR1* and *AcDHAR2*) from *Actinidia chinensis* (kiwi fruit)	Salt stress	Transgenic overexpression of these two genes (separately) in *Arabidopsis* plants was found to significantly enhance the ascorbic acid concentration and enhance the tolerance to salinity.	[185]

## 4. Significance of the Cross-Talk of the Antioxidants in Plant Biology

**Figure 6:**
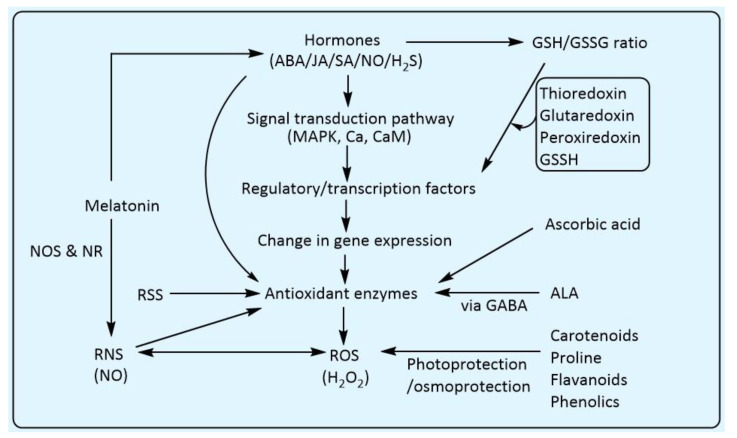
A schematic diagram of cross-talking species during functioning of antioxidant enzymes to mitigate the reactive oxygen species (ROS). Abbreviations: Abscisic acid (ABA), Jasmonic acid (JA), Salicylic acid (SA), Nitric Oxide (NO), Hydrogen sulphide (H_2_S), Mitogen-Activated Protein Kinase (MAPK), Calcium (Ca), Calmodulin (CaM), Reactive Sulphur Species (RSS), Reaction Nitrogen Species (RNS), Nitric Oxide Synthase (NOS), Nitrate Reductase (NR), 5-Aminolevulinic acid (ALA), Gamma-Amino Butyric Acid (GABA).

In plants, various mechanisms have been evolved to cope with stress conditions, and enzymatic antioxidants system plays crucial role in this challenge. Antioxidants work in close synchrony between signalling molecules of different signal transduction pathways and plant hormones (Figure 6). Initially, stress signal is perceived, after which plant synthesize hormones (salicylic acid—SA, jasmonic acid—JA, ethylene—ET and abscisic acid—ABA) according to the physiological response required to cope with the stress conditions. The synthesised hormone propagates throughout the plant system and trigger the signalling pathway to modulate changes in gene expression. Then, antioxidant enzymes are synthesised and help the plant cell to scavenge the higher level of ROS generated due to oxidative stress, thereby maintaining the redox potential, and help the plant to thrive during the stress conditions [186]. 

Cross-talk between these different components enhances the signalling coordination and ensures the stability of the plant defence system. For example, abscisic acid (ABA) is involved in multiple physiological responses in plants towards various stress conditions. Stress leads to an increase in the ABA concentration, generating a cascade of signalling events via mitogen-activated protein kinase (MAPK) cascades, and calcium and calmodulin-dependent protein kinase, which interact with H_2_O_2_ and nitric oxide (NO). Cross-talk between ROS and reactive nitrogen species (RNS) is associated by NO [187]. The ROS (H_2_O_2_) enhances the activity of the nitrate reductase leading to generation of NO, and in this way, ROS and RNS are interconnected. Accumulation of NO activates the antioxidant defence system and mitigates the harmful effect of ROS. Similarly, reactive sulphur species (such as H_2_S) also activate the SOD, CAT and APX activities and help to mitigating the oxidative damage caused by ROS. [188]. Two important signalling molecules, calcium and calmodulin (CaM), directly interact with antioxidant enzymes. Calcium directly activate CAT and GR; likewise, CaM activates CAT in the presence of Ca (Figure 6) [189]. Chloroplastic GPX is reported to influence the salicylic acid pathway. Inactivation of chloroplastic GPX in *Arabidopsis* results in a compromised photooxidative stress tolerance. However, resistance to virulent bacteria was found to be increased. Reduced expression of GPX led to higher level of ascorbic acid and salicylic acid. Furthermore, change in the leaf anatomy was also observed. This finding highlights the cross-talk of GPX in salicylic acid pathways that affect leaf development and the plant immune response [190]. More investigations are required on how chloroplastic GPX is involved in increasing the concentration of ascorbic acid and salicylic acid. Plant hormones such as ABA [191], JA [192], SA [193], and NO [194] are involved in affecting the GSH/GSSG ratio. GSH regulates the gene expression by interacting with another redox molecule, such as thioredoxin, glutaredoxin, peroxiredoxins and GSSH. Conjugation via glutationylation leads to the inactivation of thioredoxin, and de-glutationylation via glutaredoxin activates it again. The interactions between these different redox players need more investigations [195].

The role of melatonin (N-acetyl-5-methoxytryptamine) as a signalling molecule has also been illuminated in recent times, and it is known to play a significant role in cross-talk with different signalling pathways and defence mechanisms. Furthermore, melatonin also induces SA-, GA-, and ABA-dependent pathways and boosts the tolerance against several environmental stresses [196]. A methodology has been proposed for understanding the interaction between melatonin and NO, where it is reported that melatonin increase the level of NO by activating the expression of nitric oxide synthase (NOS) and nitrate reductase (NR) genes. Similarly, NO also increases the endogenous melatonin. Moreover, after a certain threshold limit, melatonin can scavenge excess NO. Both melatonin and NO increase the abiotic and biotic tolerance potential of the plant by activating antioxidant enzymes, and through the MAPK cascade signalling and SA-dependent pathway, respectively [50,197].

Cross-talk between 5-aminolevulinic acid (ALA) and antioxidant enzymes mediated by gamma-aminobutyric acid (GABA) is also reported in *S. lycopersicum*. It has been observed that exogenous application of ALA led to enhanced activity of SOD, CAT, APX and GR, thereby increasing the cold tolerance in *S. lycopersicum* plants. In this study it was found that exogenous ALA enhanced the gene expression and endogenous level of GABA. Furthermore, inhibiton of GABA with 3-mercaptopropionic (3-m p) led to reduced SOD and CAT activities, thereby decreasing the cold tolerance in *S. lycopersicum* [198].

Change in gene expression during stress-related cascading events also leads to the generation of non-enzymatic antioxidants. These non-enzymatic antioxidants help the plant in mitigating oxidative stress. For example, in drought-resistant plants, the number of carotenoid molecules per chlorophyll enhanced during stress, thus providing photoprotection from oxidative damages [199]. Drought stress induced by polyethylene glycol (20%) treatment to *O. sativa* plants led to an increase in antioxidants, such as flavonoids and phenolics, which were many folds upregulated in the stress-tolerant as compared to stress-sensitive plants [200]. Proline, an osmoprotectant as well as a sink for energy to regulate redox potentials, was found to have enhanced accumulation in water stress-tolerant cultivars than sensitive during normal and water stressed [201]. The flavonoids and proline levels were also found to be increased in salt-tolerant *O. sativa* subsp. *indica* plants compared to salt-sensitive plants, as evident by the decreased membrane damage caused by LPO [202]. The role of ascorbic acid as a non-enzymatic antioxidant has been implicated, as it is involved in the cross-talk of the defence system of plants. In an experiment, the exogenous use of ascorbic acid in *T. aestivum* plants led to the production of maximum photosynthetic pigments, photosynthetic CO_2_ assimilation rate, growth and development as compared to the normal plants subjected to water stress condition [203]. Likewise, when ascorbic acid was exogenously supplied to *S. lycopersicum* plants with and without salinity stress, it helped expedite the recovery and ensured long-term survival [204]. Ascorbic acid also assisted to relieve oxidative damage in *T. aestivum*, by enhancing leaf gas exchange and sustaining ion homeostasis [205]. The higher susceptibility of the sensitive cultivars to drought was also reflected by considerable reduction in GSH/GSSG ratio, in relation to the tolerant cultivars [200]. Ascorbic acid and GSH exhibited increased levels in the salt-tolerant cultivars when compared with the salt-sensitive cultivars [206]. Arsenic (III) significantly reduced the GSH level in *O. sativa* roots, due to its conversion to phytochelatins. The GSH demonstrated a partial plant protection during As stress, by reducing the MDA level and restoring the growth and development of As (V) stressed plants [207]. The GSH was also found to minimise the oxidative damage in *O. sativa* ultrastructure caused due to saline stress [208]. In *T. aestivum*, it was established that temperature stress induced the uptake and accumulation of GSH content and enhanced enzyme activities involved in GSH synthesis and the ratio of GSH/GSSG [209] α-tocopherol balance the cellular Na^+^/K^+^ homeostasis and hormonal balance [210]. Acute exposure of UV radiation leads to a decrease in α-tocopherol contents in plants, possibly reflecting reactions with lipid radicals [211].

## 5. Conclusions and Recommendations

The reaction mechanism of various enzymes, involved in the defence of plants against the ROS to cope-up the stress conditions, is well known. This knowledge has opened the scope of genetic engineering of important crops to achieve sustainable food security goal in an environmentally friendly way. The identification of mutants and transgenic plants with altered expression of antioxidant enzyme gene(s) is a significantly powerful approach to understand the mechanisms and functioning of the antioxidant system and its role in protecting plants against stresses. Although substantial progress has been achieved in this direction, there are still ambiguities and a lack of scientific understanding of ROS production, and how they affect plants, especially as ROS exhibit a shorter half-life and a highly reactive nature. These are the areas of future research. Direct estimation of ROS level is critical in research finding to understand the physiological state of the plant cell and to correlate it to the enzymatic activity of the antioxidant defence system. The majority of the research findings, however, lack a direct estimation of different ROS. There are few methods available for the estimation of •O2^−^ with nitro blue tetrazolium (NBT) and H_2_O_2_ by diaminobenzidine tetrahydrochloride (DAB) staining [212]. The majority of the reports mentioned the interpretation of ROS level by estimating the lipid peroxidation. Furthermore, it is also not clear which type of stress leads to how much change in different ROS. Therefore, future research needs to focus on understanding the change in different ROS under different sets of stress conditions. 

The cross-talk mechanism is not clear for the majority of the antioxidant enzymes and only superficial knowledge is available. Integration of signal sensing, hormonal synthesis and release, transduction of systemic immunity, signal transduction and increase in antioxidant enzymatic activity is lacking. The role of melatonin in such an integration is also unclear and requires further investigation. The majority of the transgenic strategies applied in different plants so far lack a holistic approach (Table 1). These studies focussed only on the expression of single genes under strong promoter, under one specific type of stress. Therefore, it is of the utmost importance that future studies investigate the gene pyramiding approach to understand the plant response to a variety of stress conditions combined together. Furthermore, the majority of the transgenic overexpression studies involved the model plant, i.e., *Arabidopsis*, so a shift in genetic engineering towards cash-crop is highly warranted to ensure the food security at a global scale. Likewise, very few transgenic studies have given information about energy reallocation and increase/decrease in the crop yield due to a change in the gene expression profile for better tolerance against stress [213]. These findings need to be elaborated more in future research endeavours. Furthermore, research is also required to identify the coding sequences to be modified through the CRISPR technique to improve the tolerance potential of the plant [214] to optimise the functioning of the native antioxidant enzymes so as to increase the enzymatic kinetics and catalytic efficiency of each of the enzymes. This will help to ensure less energy resources are being used for the defence system and more towards agriculture yield and plant biomass production. The role of non-coding RNA, microRNA and circular RNA [215] in regulating the activity of antioxidant enzymes is also understudied, and therefore, optimising the expression profile by perturbing the microRNA genes needs more investigations in the near future. 

## Figures and Tables

**Figure 3:**
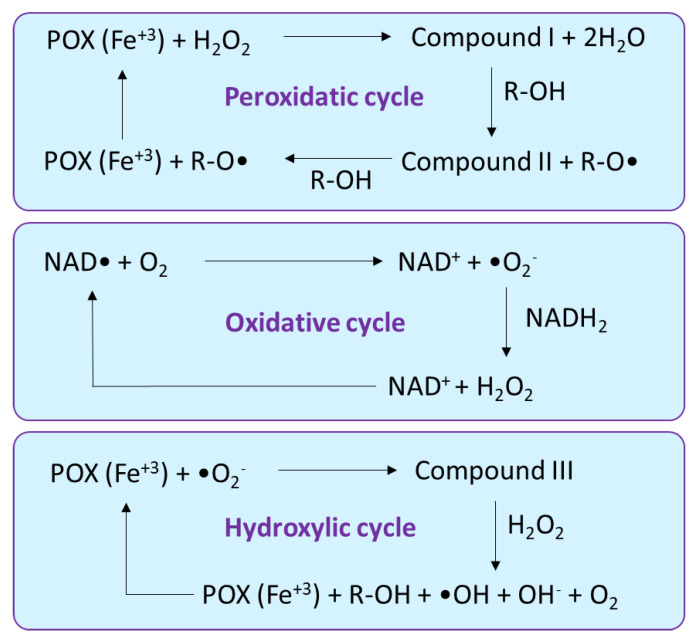
Different reaction cycles catalysed by peroxidase (POX) as reported in in vitro studies. R = any phenolic compound.

## Data Availability

No associated data marked.
